# Mother and offspring fitness in an insect with maternal care: phenotypic trade-offs between egg number, egg mass and egg care

**DOI:** 10.1186/1471-2148-14-125

**Published:** 2014-06-09

**Authors:** Lisa K Koch, Joël Meunier

**Affiliations:** 1 Department of Evolutionary Biology, Institute of Zoology, Johannes Gutenberg University of Mainz, Mainz, Germany

**Keywords:** Reproduction, Parental care, Egg cannibalism, Reciprocal causation, Cost, Insect, Earwig

## Abstract

**Background:**

Oviparous females have three main options to increase their reproductive success: investing into egg number, egg mass and/or egg care. Although allocating resources to either of these three components is known to shape offspring number and size, potential trade-offs among them may have key impacts on maternal and offspring fitness. Here, we tested the occurrence of phenotypic trade-offs between egg number, egg mass and maternal expenditure on egg care in the European earwig*, Forficula auricularia*, an insect with pre- and post-hatching forms of maternal care. In particular, we used a series of laboratory observations and experiments to investigate whether these three components non-additively influenced offspring weight and number at hatching, and whether they were associated with potential costs to females in terms of future reproduction.

**Results:**

We found negative associations between egg number and mass as well as between egg number and maternal expenditure on egg care. However, these trade-offs could only be detected after statistically correcting for female weight at egg laying. Hatchling number was not determined by single or additive effects among the three life-history traits, but instead by pairwise interactions among them. In particular, offspring number was positively associated with the number of eggs only in clutches receiving high maternal care or consisting of heavy eggs, and negatively associated with mean egg mass in clutches receiving low care. In contrast, offspring weight was positively associated with egg mass only. Finally, maternal expenditure on egg care reduced their future reproduction, but this effect was only detected when mothers were experimentally isolated from their offspring at egg hatching.

**Conclusions:**

Overall, our study reveals simultaneous trade-offs between the number, mass and care of eggs. It also demonstrates that these factors interact in their impact on offspring production, and that maternal expenditure on egg care possibly shapes female future reproduction. These findings emphasize that studying reproductive success requires consideration of phenotypic trade-offs between egg-number, egg mass and egg care in oviparous species.

## Background

The quantity and quality of offspring at egg hatching are two major components of parent and offspring fitness in oviparous species. Because these two components are traditionally thought to trade-off, females are expected to make optimal allocation decisions to maximize their reproductive success [1]. For instance, favoring the production of large clutch sizes (i.e. number of eggs) may increase the likelihood of getting a larger number of descendants. Alternatively, favoring the production of large (or massive) eggs may give rise to large offspring, which are better competitors and yield higher reproductive success than small ones [2]. Finally, spending a substantial amount of energy in egg care may enhance egg development and hatching success and may thus favor the production of numerous and / or better quality offspring [3].

Associations among egg number, egg size and egg care are generally expected to result from limited resources and life-history constraints [1,4]. For instance, limited resources have been shown to impose a trade-off between egg quantity and quality, with some females investing in large clutches of light eggs and others in small clutches of heavy eggs [2,5]. The detection of such trade-offs, however, assumes that all females within a population allocate the same quantity of resources to reproduction, which is not necessarily the case when females differ in quality (e.g. age or size) or resource acquisition [6,7]. Conversely, life-history constraints have been suggested to select for a positive association between egg size and maternal egg care [8]. One hypothesis to explain such an association is that it would allow the parents to increase the time spent with their offspring in the safest developmental stage (assuming that offspring life is hazardous and that larger eggs develop slower; safe-harbor hypothesis [9]). An alternative hypothesis is that egg mortality increases with egg weight, e.g. due to oxygen limitation in aquatic environments [6], so that these eggs require pre-hatching forms of care to develop properly [10]. To date, the ultimate reasons for the evolution of a positive association between egg size and maternal egg care are controversial, with recent comparative analyses revealing that its strength is taxon specific [11-15] and when it does occur, that its underlying evolutionary drivers are unclear ([8,10,14] but see [12]).

Although the associations between egg quantity, quality, and parental care were studied in many species [2,5,8,10,12,14], it remains unclear if and how these three parameters simultaneously shape maternal and offspring fitness at egg hatching. In general, disentangling pre- and post-laying effects on fitness returns is important for our understanding of the evolution of reproductive strategies and life-history traits [1,16], as these effects might reflect the outcome of independent selection pressures or of co-adaption processes. For instance, investment into pre-hatching forms of care may either reflect predetermined strategies of females, or serve to compensate for limited investment in the quality or quantity of eggs [17]. Here, we addressed this issue in the European earwig, *Forficula auricularia*, an insect species where females tend their clutch of eggs over winter and provide multiple forms of care, such as egg guarding, grooming and clutch displacement [18-20]. Whereas maternal attendance is required to ensure egg hatching, the frequency and duration of egg care (and thus maternal expenditure on these forms of care) are extremely variable among females [20-22]. Because earwig mothers stop feeding between egg laying and hatching [21], any change in female weight during this period can be used as a proxy to estimate its expenditure on pre-hatching forms of care. In particular, relatively high weight loss in females can be used to define high levels of maternal expenditure on egg care, whereas relatively low female weight loss (including weight gain due to egg consumption, see results) can be used to reflect low maternal expenditure on egg care. Interestingly, maternal care is not only shown towards the eggs, but also towards the young offspring (called nymphs) after hatching. In particular, mothers stay with their nymphs for several weeks during which they provide multiple forms of care, such as protection against predators and food provisioning [18,19]. Although post-hatching care is known to come with substantial costs for the mothers - for example by delaying their 2^nd^ clutch production [18] - the potential costs of pre-hatching care remain unknown in this species. Finally, the size of earwig offspring at hatching is particularly important, as it generally enhances nymph survival and limits the risks of cannibalism after brood mixing, a common phenomenon during which nymphs join unrelated clutches [23,24]. Once the period of family life has ended, mothers disperse and some produce a second and final clutch [19].

We investigated the associations between egg number, egg mass and maternal expenditure on egg care, as well as their simultaneous influence on maternal and offspring fitness at egg hatching. We first surveyed a total of 80 clutches to (1) determine the occurrence of trade-offs among these three parameters, (2) test whether variation in female condition (body weight) possibly masks these trade-offs and more generally (3) investigate whether clutch size, egg size and maternal expenditure on egg care additively or interactively combine to determine offspring number and weight at egg hatching. In the case of interactions, we predict that egg number only determines nymph number and weight when mothers also express high investment into egg care (i.e. higher weight loss between egg laying and egg hatching). We then set up a mother-removal experiment using 40 clutches to test whether (4) low maternal expenditure on egg care can be beneficial to the female, e.g. in terms of 2^nd^ clutch production, and whether (5) these benefits remain significant after females interact with their hatched nymphs [18,19,22,25].

## Results

**Figure 1:**
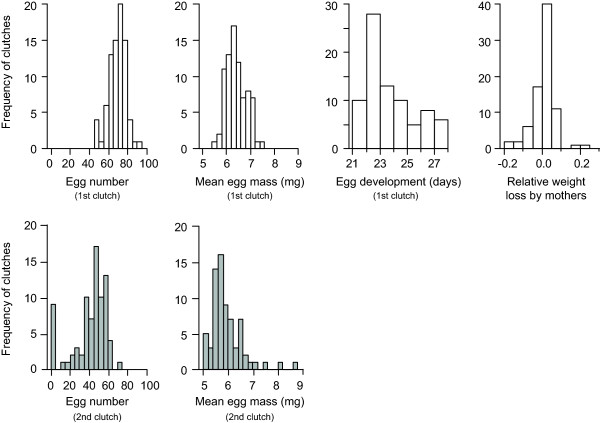
Distribution of the traits measured in first (white) and second (grey) clutches.

The 1^st^ clutches produced by the 80 females contained between 47 and 91 eggs, which weighed between 0.55 and 0.75 mg on average and hatched between 21 and 28 days after they had been laid (Figure 1). The relative weight change of females during the period of egg care was highly variable, ranging from a 22.2% loss to a 16.8% gain of the initial weight. Note that maternal weight gain likely reflects egg consumption, as females had no access to food during the period of egg care. Most females (53, i.e. 66.3%) lost weight during the period of egg care (Figure 1), so that weight loss was used as a reference for weight change in the rest of the study (i.e. positive weight change stands for female weight loss and negative values for weight gain). In the mother-removal experiment, 18 out of the 20 isolated and 15 out of the 20 non-isolated females produced a 2^nd^ clutch (Fisher Exact test, P = 0.408), which contained between 15 and 74 eggs, weighing between 0.50 and 0.87 mg on average (Figure 1). These 2^nd^ clutches were overall smaller (paired t-test on egg number, t = 8.73, d.f. = 39, P < 0.0001) and lighter (paired t-test on mean egg mass, t = 2.65, d.f. = 32, P = 0.013) than the 1^st^ ones.

Overall, there was no association between egg number, mean egg mass and the relative weight loss by mothers during the period of egg care (Table 1A). However, when correcting for variation in female weight at egg laying, the residuals of egg number were negatively correlated with both the residuals of mean egg mass (Table 1B) and the relative weight loss during egg care (Table 1B). Although heavy or large eggs are known to need more time to develop across species [9], we found that egg developmental time was independent of mean egg mass, of the number of eggs or of female expenditure on egg care (Table 1A and 1B).

**Table 1:** Associations of egg number, egg mass, maternal expenditure on egg care and egg developmental time

	**(A) Uncorrected values**					**(B) Corrected values**			
	**Egg number**	**Mean egg mass**	**Mother w loss**	**Egg dvpt**		**Egg number**	**Mean egg mass**	**Mother w loss**	**Egg dvpt**
Egg number	X	0.11	−0.20	−0.1		X	**−0.28**	**−0.24**	−0.01
Mean egg mass	0.321	X	−0.20	−0.08		**0.013**	X	−0.21	−0.05
Mother w loss	0.069	0.075	X	−0.09		**0.032**	0.066	X	−0.09
Egg dvpt	0.370	0.488	0.431	X		0.958	0.651	0.431	X

Maternal expenditure on egg care was estimated through the relative weight loss by mothers between egg laying and egg hatching. This correlation matrix was conducted using (A) uncorrected values or (B) the residuals of egg number and mean egg mass (i.e. values corrected for female weight at egg laying). The matrices above diagonal reports the correlation coefficient and the ones below diagonal the corresponding P-values, both obtained from Spearman rank correlation tests. The correlations significant after false discovery rate (FDR) corrections are in bold (see text).

**Figure 2:**
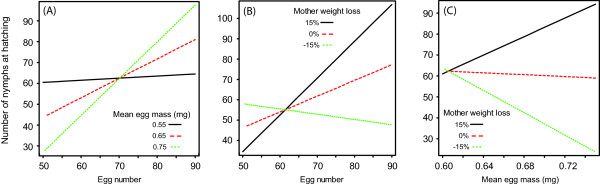
**Interacting effects of egg number, egg mass and egg care on nymph number.** The number of nymphs produced at hatching resulted from interactions between **(A)** egg number and mean egg mass, **(B)** egg number and mother weight loss and **(C)** mother weight loss and mean egg mass. As an illustration, the regressions lines are given for when **(A)** the mean egg mass was 0.55 mg (black), 0.65 mg (red) and 0.75 mg (green), as well as when **(B & C)** the relative female weight loss was 15% (black), 0% (red) or −15% (green).

**Figure 3:**
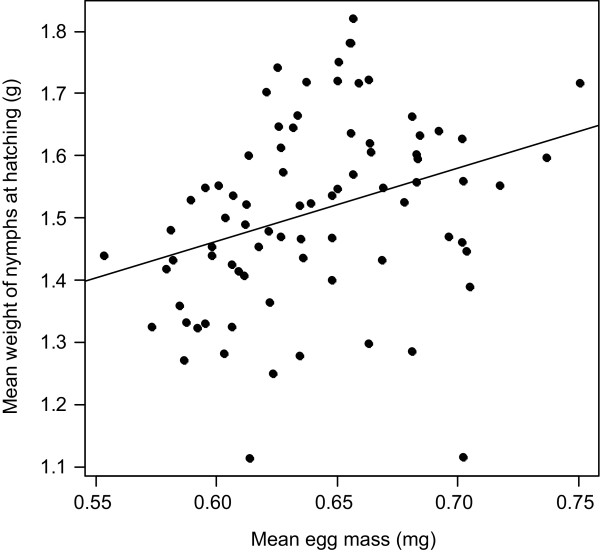
Correlation between mean egg mass and mean weight of nymphs at hatching.

A series of pairwise interactions among egg number, mean egg mass and female weight loss determined the number of hatched nymphs (Table 2A), a result supporting the entangled effects of maternal expenditure on egg care and egg production on nymph production. In particular, decreases in mean egg mass or in relative weight loss by mothers during egg care cancelled the otherwise positive association between egg and nymph numbers (Table 2A, Figures 2A and 2B). Conversely, decreases in the weight loss by mothers entailed a negative association between mean egg mass and nymph number (Table 2A, Figure 2C). Independently from the interactive effects on nymph number presented above, the mean weight of nymphs at hatching was positively associated with the mean egg mass (Table 2B, Figure 3), but independent of egg number, mother weight loss or any interaction among the three tested factors (Table 2B). Overall, nymph number was independent of the mean weight of nymphs at hatching (Spearman rank correlation test; r_s_ = −0.15, S = 81049, P = 0.190).

**Table 2:** Influences of egg number, egg mass and maternal expenditure on egg care on (A) nymph number and (B) mean nymph weight

	**(A) Nymph number**				**(B) Mean nymph weight**		
	**LR χ**^**2**^	**d.f.**	**P**		**LR χ**^**2**^	**d.f.**	**P**
Egg number (EN)	56.07	1	**< 0.0001**		0.26	1	0.607
Mean egg mass (MEM)	0.05	1	0.816		8.78	1	**0.003**
Mothers weight loss (MWL)	7.78	1	**0.005**		0.35	1	0.552
EN : MEM	7.71	1	**0.005**		0.01	1	0.938
EN : MWL	11.66	1	**0.001**		1.18	1	0.277
MEM : MWL	7.27	1	**0.007**		0.01	1	0.921

Maternal expenditure on egg care was estimated through the relative weight loss by mothers between egg laying and egg hatching. Significant P-values are in bold.

**Figure 4:**
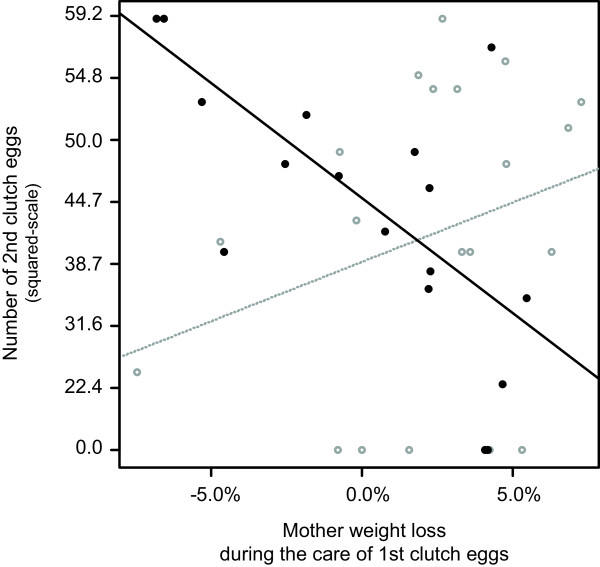
**Influence of maternal expenditure on egg care on their 2**^**nd **^**clutch.** Maternal expenditure on egg care was estimated through their relative weight loss between egg laying and egg hatching. After hatching of their 1^st^ clutch eggs, females experienced (grey) or did not experience (black) post-hatching family life.

The relative weight loss of mothers during egg care affected their investment into future reproduction, but this effect depended on the occurrence of post-hatching family life (GLM; Interaction between relative mother weight loss and occurrence of post-hatching family life: Likelihood ratio (LR) χ^2^ = 11.33, d.f. = 1, P = 0.0008). In particular, mother weight loss was negatively correlated with the number of 2^nd^ clutch eggs when mothers were isolated from their 1^st^ clutch nymphs at egg hatching (Figure 4; GLM estimate ± SE = −138.8 ± 73.4, t = −3.58, P = 0.001) but not when they were kept with their nymphs after hatching (GLM estimate ± SE = − 93.6 ± 80.8, t = 1.34, P = 0.188). In contrast, the mean egg mass of the 2^nd^ clutch was independent of the three components of reproductive success measured on the 1^st^ clutch and the occurrence of family life (GLM; Relative mother weight loss during 1^st^ clutch: LR χ^2^_1_ = 0.30, d.f. = 1, P = 0.583; Mean egg mass measured in the 1^st^ clutch: LR χ^2^_1_ = 0.36, d.f. = 1, P = 0.546; Post-hatching family life: LR χ^2^_1_ < 0.01, d.f. = 1, P = 0.924; Interactions, all P > 0.384).

## Discussion

Although egg number, egg mass and egg care are the three most common components of reproductive success in oviparous females, the reciprocal influences among them and the consequences of their joint action on maternal and offspring fitness remain under debate. Here we showed that in the European earwig *F. auricularia* (1) egg number, egg mass, expenditure on pre-hatching forms of care and egg developmental time are independent of each other. However, (2) when correcting egg mass and egg number for natural variation in female weight at egg laying, our data revealed a trade-off between these two parameters, as well as a trade-off between egg number and pre-hatching care. We also demonstrated that (3) the number of hatching nymphs does not only depend on egg number, but also on the mean egg mass and on the level of maternal investment into egg care. In particular, low maternal expenditure on egg care led to a negative association between mean egg mass and nymph number, as well as weakened positive association between egg and nymph numbers. Moreover, clutches of light eggs were less likely to hatch than clutches of heavy ones. Independent of these effects on nymph number, our data showed that (4) the mean weight of nymphs at hatching was positively associated with the mean egg mass, but independent from egg number and investment into pre-hatching care. Finally, our results revealed that (5) maternal expenditure on the care of 1^st^ clutch eggs reduced the production of 2^nd^ clutch eggs, but only in absence of post-hatching family interactions.

Variation in the amount of resources available to each individual is traditionally expected to mask potential investment trade-offs between mutually exclusive functions [26-28]. Our findings support this prediction in *F. auricularia*. Here, we show that correcting egg number and mean egg mass by female weight at egg laying allowed the detection of negative associations between egg quantity and quality and between egg quantity and egg care. The negative association between egg number and mean egg mass reflects a traditional trade-off found across a wide range of species [4,26-28], which shows females’ needs to distribute resources among two traits simultaneously expressed at egg laying. Conversely, the negative association between egg number and egg care involves two temporally separated traits, which demonstrates that maternal expenditure on egg care is not a fixed strategy before egg production, but instead determined by females’ clutch size. We propose four hypotheses to explain the reported level of maternal expenditure on pre-hatching forms of care. First, this level may simply be a by-product of the resources left to females directly after egg production, so that females are energetically constrained in their level of care [1]. The importance of resource availability on the level of (post-hatching) care, however, received mixed support across species (e.g. [19,29-31]). Second, it may reflect an adaptive strategy of the females which favors investment into either egg number or pre-hatching care [16]. For instance, low risks of egg predation and thus low benefits of egg guarding could favor females allocating more resources into egg number than egg care [32]. A third hypothesis is that the level of maternal expenditure on pre-hatching care might not be directly affected by egg production, but by the impact of egg production on egg quality. In other words, clutches where the number of eggs was higher than predicted by female weight could have required (or triggered) the expression of higher levels of care. Disentangling between these first three hypotheses would require cross-fostering of eggs and females [33], as well manipulation of clutch size to then investigate whether the level of maternal expenditure on egg care is determined by the number of eggs produced or tended by the female, as well as by the origin of the tended eggs.

The fourth hypothesis to explain the negative association between egg number and maternal expenditure in egg care is that larger egg production entailed larger egg consumption by females. Maternal expenditure on egg care was estimated by measuring mother weight loss between egg laying and hatching, a period in which females had no access to food. Although this measurement may at least partly reflect energy expenditure [19,20], the fact that 27 (33.8%) females gained weight during this period of time implicates that at least some mothers consumed a few of their eggs. Filial egg consumption has never been reported in the European earwig (but it was seen in the maritime earwig, *Anisolabis maritima*[34]), but cannibalism frequently occurs in this species, either of eggs by newly hatched nymphs, between siblings during family life or even between adults during group living [21,35,36]. More generally, filial egg consumption is a behavior found in many species exhibiting parental care [37]. This behavior may reflects either (1) a stress-induced behavior with limited evolutionary relevance or (2) an adaptive strategy of females, which may serve to recycle resources from current eggs for future reproduction or into higher quality of care for the current clutch, as well as to limit the level of sibling competition after hatching [38-40]. In *F. auricularia*, the occurrence of filial egg consumption is unlikely to reflect a stress-induced behavior as all females were maintained under standard laboratory conditions that are typically associated with very high hatching success (e.g. [19,22,41]). Conversely, the benefits of low expenditure into pre-hatching care (and possibly of higher egg consumption) in terms of 2^nd^ clutch production are in line with filial egg cannibalism as an adaptive strategy of earwig females. Further studies should investigate whether filial egg cannibalism results from the targeted consumption of non-viable (trophic eggs [3,42]) or viable eggs, and determine whether such behavior was selected to help females re-allocating resources toward future reproduction and/or limiting the level of future sibling competition.

Our data reveals both isolated and interactive effects of maternal expenditure on egg care, clutch size and egg weight on offspring weight and offspring number at egg hatching. On one hand, nymph weight was positively associated only with egg mass, a finding in line with those in other oviparous species [2,43]. On the other hand, nymph number was neither shaped by single nor additive effects among the three parameters, but by pairwise interactions. In particular, nymph number was positively associated with egg number only in clutches with heavy eggs or where mothers lost more weight during egg care, but also negatively associated with mean egg mass in clutches where mothers gained weight during egg care. If change in female weight at least partly reflects egg consumption, this negative association suggests that *F. auricularia* mothers preferentially fed on clutches of large eggs. Moreover, the overall negative association between mother weight gain and nymph number supports that female weight change during egg care is a good proxy to estimate the efficiency of maternal pre-hatching care (including the costs of egg consumption) on hatching success. Finally, the trade-off between egg mass and nymph number together with the positive association between egg mass and nymph weight reveals fitness costs and benefits of producing heavy eggs, but also shows that these costs can be limited when mothers subsequently invest into pre-hatching care (or limit egg consumption). Hence, maximizing fitness returns for earwig mothers and nymphs requires the simultaneous investments in heavy eggs and egg care. On the intra-species level, this finding is in line with comparative studies reporting the positive association between egg size and parental care across species and taxa [8,10-15]. More generally, it also supports that the sensitivity of heavy eggs to pre-hatching care could be a key driver in the emergence of such an association [12].

Lower weight loss (and weight gain) by mothers during the care of 1^st^ clutch eggs translated into a higher number of eggs in the 2^nd^ clutch, which revealed that low expenditure on egg-care (including high levels of egg consumption) provided benefits to females in terms of future reproduction. However, this association was only detectable when mothers were isolated from their hatchlings, suggesting that family life cancelled the maternal benefits of low expenditure on egg care and/or provided benefits to females with high expenditure on egg care, both in terms of 2^nd^ clutch egg number. Whereas the second hypothesis requires forms of cooperation from nymphs to mothers, which to our knowledge have never been reported in species with family life, the first one could reflect a trade-off between pre- and post-hatching care, with females expressing low expenditure on egg care (including higher egg consumption rates) subsequently showing high levels of offspring care, and vice-versa. Because we did not measure the level of post-hatching care in this experiment, our data does not allow directly testing the occurrence of such a trade-off, or the ultimate reasons for its evolution (by-product or active strategy). However, this trade-off would be in line with the high variation in the levels of post-hatching care already described in *F. auricularia* females, e.g. in terms of food provisioning, aggressive protection against predators, clutch displacement and allogrooming [18,19,22,25]. More generally, this result raises the question of the importance of pre-hatching parameters and post-hatching family life on offspring fitness. For example, post-hatching maternal care has been shown to mask the otherwise positive effects of egg size on larval body mass at dispersal in the burying beetle *Nicrophorus vespilloides*[44]. Notice however that in the burying beetle females do not provide pre-hatching forms of care, which might have entailed different selection pressures on the respective importance of egg number and size, as well as pre- and post-hatching care between the two species.

## Conclusions

In this study, we have shown phenotypic reproductive trade-offs between egg number, egg mass and pre-hatching care, as well as demonstrated their interactive effects on maternal reproductive success. We also demonstrated that studying natural variation in female body weight at egg laying is of key importance to better understand female investment trade-offs, as this variation masked the trade-off between the quantity and quality of eggs (a key trade-off reported in other species, e.g. [6,7,43]) and the one between egg quantity and maternal egg care. Moreover, our data suggests that pre-hatching care is costly for mothers in terms of future reproduction, but that these costs could be compensated by lower investment in post-hatching care. Overall, these results emphasize that studying fitness returns of oviparous mothers and offspring requires considering reciprocal influences among the multiple types of maternal investments at egg production, and more generally support the very recent claim to incorporate reciprocal causation in evolutionary theory [45].

## Methods

A total of 80 females and 73 males of the European earwig, *F. auricularia*, were collected in September 2012 in Dolcedo, Italy. The individuals were then transferred to three plastic containers of comparable group size (balanced sex-ratio; 37 × 22 × 25 cm) and maintained for one month under standard laboratory conditions (12:12 h day:night, 20:18°C and constant 60% humidity). Each container was furnished with humid sand, egg cardboards and *ad libitum* food that was changed twice a week (see food composition in [19]). One month later, females were isolated in Petri dishes (10 cm diameter) to enable egg production [19]. The Petri dishes contained humid sand as a substrate, a plastic shelter as a nest and were maintained under complete darkness at 15°C and 60% humidity. Each female received *ad libitum* food until egg laying. Females were checked on a daily basis to record the first days of egg laying and egg hatching, and thus to calculate the egg developmental time (in days). Because eggs are generally laid within three days and hatch within one day, the number of eggs was counted three days after the first egg laying and the number of nymphs one day after the first egg hatching. On the days of counting, we weighed a group of ten randomly chosen eggs or ten randomly chosen nymphs per family and divided the values by 10 to obtain the mean egg mass and mean nymph weight, respectively. The relative weight loss by females during the period of egg care was measured by subtracting female weight at egg hatching from female weight at egg laying and dividing this value by female weight at egg laying. Maternal expenditure on egg care was defined as female weight loss during the period of egg care, because *F. auricularia* females (1) do not forage from egg laying to egg hatching [21], (2) lose weight due to their expression of energetically costly forms of care [19] and (3) may only gain weight due to filial egg consumption [34] so that negative female weight loss still reflects an extreme form of low expenditure on egg care. Note that all results remain qualitatively the same when using the absolute instead of the relative weight loss by females. All weighing was done to the nearest 0.001 mg using a micro scale (Pescale MYA 5).

We then investigated whether (1) the level of maternal care towards 1^st^ clutch eggs affected their investment into 2^nd^ clutch production and (2) whether post-hatching family life possibly masks such an association. To this end, 40 clutches were randomly sampled out of the 80 mentioned above (there was no difference between the two subsets regarding all the measured traits; MANOVA using egg number, mean egg mass, egg developmental time, number of nymphs at hatching, mean weight of nymphs at hatching, relative weight loss by mothers, mother weight at egg laying; Approx. F_7,72_ = 0.91, P = 0.501), while the other clutches were used in a different experiment (not presented here). Out of the 40 clutches, 20 mothers were isolated in new Petri dishes (diameter 10 cm) one day after their 1^st^ clutch eggs hatched and 20 were first transferred to new Petri dishes with their 1^st^ clutch nymphs for 16 days (under standard laboratory conditions, see [19]) and then isolated in new Petri dishes (diameter 10 cm). These two groups of 20 females did not differ regarding the above measured traits (MANOVA, Approx. F_7,32_ = 0.82, P = 0.578). Isolated mothers were then maintained under standard laboratory conditions (see above), received food twice a week and were checked on a daily basis to record 2^nd^ clutch production. Seven out of the 40 females did not produce a 2^nd^ clutch 60 days after their isolation and were thus considered as one clutch producers (i.e. the number of 2^nd^ clutch eggs get the value 0, [19]). Like in the 1^st^ clutch measurements, the number of eggs produced in the 2^nd^ clutch and their mean mass were measured three days after the first egg has been observed. All experiments comply with European laws.

A series of spearman rank correlation tests was conducted to test for potential associations between the relative weight loss by mothers during egg care, egg number and mean egg mass. Because a key and common assumption in literature on maternal investment into egg quality is that large eggs take more time to develop [9], we also included egg developmental time in the correlation matrix. To determine whether female condition possibly masks trade-offs in the measured traits, we then re-ran the above correlation tests using the egg number and mean egg mass corrected for female weight at egg laying. This correction was obtained by extracting the residuals from two linear models in which the female weight at egg laying was used as explanatory variable and either the egg number (r = 0.54, F_1,73_ = 30.8, P < 0.0001) or the mean egg mass (r = 0.48, F_1,73_ = 21.6, P < 0.0001) as response variable. The significance level α = 0.05 in the correlation tests was adjusted for multiple testing to α = 0.033 using the false discovery rate method [46]. Two Generalized Linear Models (GLM) were then used to test whether egg number, mean egg mass, relative weight loss by mothers and their interactions influenced the number and the mean weight of nymphs at hatching. Finally, two GLMs were fitted to test whether maternal expenditure on 1^st^ clutch care influences female investment into future reproduction. A first GLM was run using the relative female weight loss during egg care, the number of eggs produced in the 1^st^ clutch, the occurrence of post-hatching family life and their interactions as explanatory variables and the square-transformed number of eggs produced by females in their 2^nd^ clutch as response variable. The second GLM was run using the same explanatory variables, but with the mean egg mass of the 2^nd^ clutch eggs as response variable. All statistical analyses were conducted using the software *R* 3.0.2. Interactions between continuous factors were plotted using the package *effects*, which display the predicted values of a given GLM while controlling for the values in one of the interacting variables (details in [47]).

### Availability of supporting data

The data set supporting the results of this article is available in the DRYAD repository, http://doi:10.5061/dryad.p9t05[48].

## Competing interests

The authors declare that they have no competing interests.

## Author’s contributions

LKK and JM conceived and designed the experiments. LKK carried out the experiments. JM conducted the statistical analyses and wrote the paper. All authors read and approved the final manuscript.
